# Intrinsic Balance between ZEB Family Members Is Important for Melanocyte Homeostasis and Melanoma Progression

**DOI:** 10.3390/cancers12082248

**Published:** 2020-08-11

**Authors:** Kenneth Bruneel, Jeroen Verstappe, Niels Vandamme, Geert Berx

**Affiliations:** 1 Molecular and Cellular Oncology Laboratory, Department of Biomedical Molecular Biology, Ghent University, Technologiepark-Zwijnaarde 71, 9052 Ghent, Belgium; kenneth.bruneel@ugent.be (K.B.); jeroen.verstappe@ugent.be (J.V.); niels.vandamme@irc.vib-ugent.be (N.V.); 2 Cancer Research Institute Ghent (CRIG), Ghent University, 9000 Ghent, Belgium; 3 VIB-UGent Center for Inflammation Research, Ghent University, Technologiepark-Zwijnaarde 71, 9052 Ghent, Belgium

**Keywords:** ZEB transcription factors, melanoma, melanocyte development and homeostasis, phenotype switching, tumour heterogeneity, cellular plasticity

## Abstract

It has become clear that cellular plasticity is a main driver of cancer therapy resistance. Consequently, there is a need to mechanistically identify the factors driving this process. The transcription factors of the zinc-finger E-box-binding homeobox family, consisting of ZEB1 and ZEB2, are notorious for their roles in epithelial-to-mesenchymal transition (EMT). However, in melanoma, an intrinsic balance between ZEB1 and ZEB2 seems to determine the cellular state by modulating the expression of the master regulator of melanocyte homeostasis, microphthalmia-associated transcription factor (MITF). ZEB2 drives MITF expression and is associated with a differentiated/proliferative melanoma cell state. On the other hand, ZEB1 is correlated with low MITF expression and a more invasive, stem cell-like and therapy-resistant cell state. This intrinsic balance between ZEB1 and ZEB2 could prove to be a promising therapeutic target for melanoma patients. In this review, we will summarise what is known on the functional mechanisms of these transcription factors. Moreover, we will look specifically at their roles during melanocyte-lineage development and homeostasis. Finally, we will overview the current literature on ZEB1 and ZEB2 in the melanoma context and link this to the ‘phenotype-switching’ model of melanoma cellular plasticity.

## 1. Introduction

To date, melanoma is the most severe cutaneous cancer. Its incidence rate is increasing and it has the highest mortality rate of all skin cancers [1]. Melanomas arise from melanocytes in which mutations typically target the proteins of the mitogen-activated protein kinase (MAPK) signalling pathway: BRAF (viral RAF murine sarcoma viral oncogene homolog B1) and NRAS (neuroblastoma RAS viral oncogene homolog). Indeed, BRAF^V600E^ and NRAS^Q61K^ mutations are the main oncogenic drivers in nearly 85% of human melanomas. Melanocyte differentiation and proliferation are dependent on MAPK signalling, which is tightly controlled by the binding of growth factors, such as stem cell factor (SCF), basic fibroblast growth factor (bFGF) and hepatocyte growth factor (HGF), to heterodimeric G protein-coupled receptors (GPCRs) and receptor tyrosine kinases (RTKs). However, in melanoma cells harbouring BRAF^V600E^ or NRAS^Q61K^ mutations, this signalling pathway is constitutively active, which results in uncontrolled cell growth [2].

Current therapies consist mainly of combined targeted therapies against this signalling pathway using a combination of inhibitors of BRAF and MEK (mitogen-activated protein kinase kinase 1) or a combination of BRAF inhibitors and immunotherapies, e.g., the use of anti-CTLA4 (cytotoxic T-lymphocyte associated protein 4) or anti-PD1 (programmed cell death protein 1) (recently reviewed in [3,4]). The overall survival rate of melanoma patients has increased due to these novel therapeutic options [1]. Nevertheless, intrinsic and acquired resistance in melanoma cells still impede effective treatment of a subgroup of melanoma patients.

An important mechanism driving this resistance is tumour heterogeneity. As different subpopulations are present in a tumour, some of them respond to the current therapies while others find a way to escape and cause remission. Single cell analyses currently allow characterising these melanoma subpopulations and identifying the signalling pathways modulating melanoma heterogeneity. It is becoming clear that a combination of (epi)genetic modifications, micro-environmental cues and reversible phenotypic changes within the melanoma cells are responsible for cellular plasticity. It appears that an intrinsic balance of the zinc-finger E-box-binding homeobox family of transcription factors (ZEBs) plays a central role in this melanoma cell plasticity. Moreover, to understand the mechanisms by which these ZEBs function, this review first provides an overview of how these transcription factors generally function and looks specifically at their roles during melanocyte lineage development and homeostasis. Furthermore, we describe the “phenotype-switching” model of melanoma cell plasticity and summarise the current data on the ZEB transcription factors in this melanoma progression model.

## 2. The ZEB Transcription Factors Family

### 2.1. ZEB1 and ZEB2 Homology and Diversification

**Figure 1:**
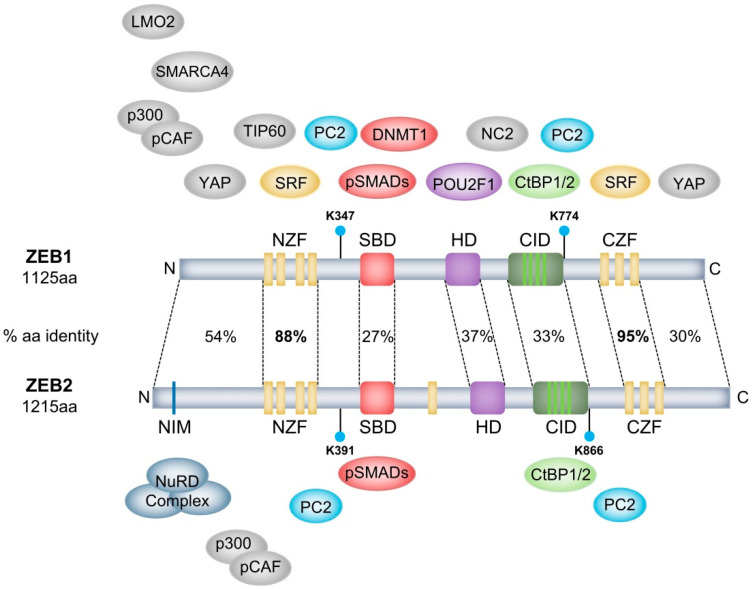
Human ZEB1 and ZEB2 protein structure and interaction partners. Percentages depict the amino acid (aa) sequence similarity. Only interaction partners of which the binding site on ZEB1/ZEB2 has been studied are depicted. For a full list of all interaction partners, see Table 1 and Table 2. Interaction partners have an identical colour as their respective binding domain on ZEB1/ZEB2. PC2 (in blue) binds at and sumoylates Lys-347 and Lys-774 of ZEB1 and Lys-391 and Lys-866 of ZEB2 (cyan circles). Brown/Yellow coloured interaction partners have interaction sites that are defined as bigger region. The NuRD complex is a multi-protein complex of which the exact interaction partner with ZEB2 is not defined.

The ZEB family of transcription factors consists of two proteins, ZEB1 and ZEB2. These are highly modular proteins containing different well-defined domains that are important for DNA-binding or protein–protein interactions with other transcription factors or co-factors (Figure 1). ZEB1 and ZEB2 are characterised by two highly homologous clusters of zinc fingers separated by a homeodomain [5]. In both proteins, the N-terminal zinc-finger cluster (NZF) contains four zinc fingers (three CCHH fingers and one CCHC finger) and the C-terminal zinc-finger cluster (CZF) contains three CCHH zinc fingers. These clusters can bind independently to bipartite E-box (5′-CANNTG) or E-box-like DNA motifs [6,7]. Except for human ZEB1, most ZEB1/ZEB2 orthologues contain a single additional zinc finger (ZF) upstream of the central POU-like homeodomain (HD). In contrast to most HDs seen in other proteins, in ZEB transcription factors, this domain does not seem to bind DNA. However, the rat ZEB1 HD is important for protein–protein interactions, as shown by its interaction with the transcription factor POU2F1 (POU class 2 homeobox 1) [8]. Vertebrate ZEB1 and ZEB2 share up to 85% protein sequence identity at the zinc-finger clusters. However, other domains within these proteins share as little as 30–50% sequence identity [7]. This indicates that ZEB1 and ZEB2 share common DNA-binding specificities and target genes, yet they evolved to interact with diverse interaction factors. In this way, they can partly perform overlapping functions in some contexts while performing specific and independent protein–protein interactions in others.

This functional diversification is also present at the genomic level. The 5′ untranslated region (5′ UTR) of *Zeb2* (but not *Zeb1*) is highly complex, consisting of nine untranslated exons upstream of the first translated exon. Alternative splicing of diverse combinations of these exons generates multiple splice variants of *Zeb2*. This is modulated by three different transcription initiation sites in the 5′UTR in a tissue-specific manner [9]. How these splice forms affect ZEB2 functionality is not known and it would be interesting to see how these ZEB2 variants determine their specific expression patterns and functions in different tissues. Moreover, a large intron in the 5′UTR of *Zeb2* harbours an internal ribosomal entry site (IRES) that promotes proper translation of ZEB2. SNAIL (Snail family transcriptional repressor 1) induces the expression of a *Zeb2* antisense transcript (*Zeb2-AS*) that overlaps the 5′ splice site in this intron. In this way, SNAIL-induced *ZEB2-AS* expression prevents splicing of this intron, thereby retaining the IRES and increasing ZEB2 expression [10].

### 2.2. ZEB1 and ZEB2 Interact with the CtBP1/2 Co-Repressor Complex

Both proteins were originally identified as transcriptional repressors and are still mainly considered as such. ZEB1, initially named δEF-1, was first reported to be important during chicken lens development as a transcriptional repressor of the δ1-crystalline enhancer [11]. ZEB2, also known as SIP1, was identified as an interaction partner of the MH2 domain of different SMAD proteins (mothers against decapentaplegic homolog) of the BMP (bone morphogenetic protein) and TGF-β (transforming growth factor-β) pathways in *Xenopus*. This complex can bind and repress the *Xbra2* promoter, pointing to a role in mesodermal differentiation [7]. Both ZEB1 and ZEB2 can bind to the co-repressors C-terminal binding protein 1 and 2 (CtBP1/2) via their CtBP-interacting domain (CID) (Figure 1). This domain consists of multiple PXDLS motifs that are crucial for this interaction [12,13]. Upon binding to ZEB1/2, CtBP1/2 recruit a co-repressor core complex consisting of histone deacetylases 1 and 2 (HDAC1/2), euchromatic histone lysine methyltransferases 1/2 (EHMT1/2), the chromodomain-containing proteins histone promoter control protein 2 (HPC2) and chromodomain Y-like (CDYL), REST corepressor 1 (CoREST), ligand-dependent nuclear receptor corepressor (LCOR), lysine-specific demethylase 1 (LSD1), ubiquitin-conjugating enzyme E2I (UBC9) and polycomb protein 2 (PC2) [14,15,16]. These ZEB1/2-CtBP1/2 repressor complexes are important for downregulating the epithelial marker E-cadherin (CDH1) during carcinoma progression [17,18,19] and during developmental processes such as neurulation and pituitary lactotrope differentiation [13,15]. A role for ZEB2–LSD1 interaction has also been identified in T-cell acute lymphoblastic leukaemia (T-ALL) [20] and a ZEB1–HDAC1/2 interaction was shown to downregulate CDH1 in pancreatic cancer [21]. This suggests a role for the CtBP1/2 co-repressor complex in these contexts. However, as the presence of multiple components of the CtBP1/2 co-repressor complex has not been defined experimentally yet, it is still possible that the ZEB2–LSD1 and ZEB2–HDAC1/2 interactions are present as part of another co-repressor complex.

### 2.3. CtBP1/2-Independent Transcriptional Repression via ZEB1 and ZEB2 

The ZEB transcription factors can also repress target genes independently of CtBP1/2. CID-mutated ZEB1 and ZEB2 proteins were still able to repress CDH1 [22]. ZEB1, for example, can repress CDH1 expression by recruiting the SWI/SNF (switch/sucrose non-fermentable) chromatin-remodelling protein named SWI/SNF-related, matrix-associated, actin-dependent regulator of chromatin subfamily A member 4 (SMARCA4) [23]. In mammary gland and breast carcinoma, ZEB1 inhibits CDH1 expression by interacting with DNA methyltransferase 1 (DNMT1) [24]. A complex of ZEB1 and human telomerase reverse transcriptase (hTERT) associates with and represses the *CDH1* promoter, driving epithelial-to-mesenchymal transition (EMT) in colorectal cancer [25]. That study and others have illustrated that hTERT drives carcinogenesis not only by maintaining telomere length, but also by acting as a transcriptional regulator of various cancer-associated processes [26]. Furthermore, two articles describe CDH1 repression in prostate cancer by ZEB1 binding either to NAD-dependent protein deacetylase sirtuin-1 (SIRT1) or to lysine methyltransferase 5A (SET8) [27,28].

Besides its repression of CDH1, ZEB1 can repress CD4 in Jurkat cells by interacting with Tat-interacting protein 60 (TIP60) [29]. In vitro, ZEB1 binds to negative cofactor 2 (NC2), which is an inhibitor of RNA polymerase II and III [30]. Less is known about the interaction partners of ZEB2. Nevertheless, it has been shown that ZEB2 directly interacts with the nucleosome remodelling/histone deacetylating (NuRD) co-repressor complex at its N-terminally located NuRD complex interacting motif (NIM) (Figure 1). ZEB2 haploinsufficiency has been established as a driver of the Mowat-Wilson syndrome in a subgroup of patients. Deletion of this NIM domain of ZEB2 was found to result in a mild form of Mowat–Wilson syndrome [31]. More recently, a ZEB1–NuRD complex was also shown in lung cancer. This complex suppresses TBC1 domain family member 2b (TBC1D2b), stimulating internalisation of CDH1. Unlike for ZEB2, it was not determined which domain of ZEB1 interacts with the NuRD complex [32].

### 2.4. ZEB1 and ZEB2 as Transcriptional Activators 

The ZEB transcription factors can also function as transcriptional activators. ZEB1 can interact with serum response factor (SRF) via the NZF and CZF domains. Together with SMAD3, this complex induces differentiation of smooth muscle cells by activating the smooth muscle α-actin promotor [33]. Next to this, interaction with the hematopoietic regulator LIM only domain 2 (LMO2) inhibits ZEB1-mediated activation of matrix metalloproteinase-1 (MMP-1) and expression of vascular endothelial growth factor (VEGF) [34]. In breast cancer cells, it has been shown that yes-associated protein 1 (YAP) recruits ZEB1 and turns it into a transcriptional activator, upon which they drive a common ZEB1/YAP target gene set [35]. While ZEB1/2 interact with PC2 as part of the CtBP co-repressor complex, binding of PC2 to ZEB1/2 can also result in sumoylation at lysine-347 and lysine-774 in ZEB1 and lysine-391 and lysine-866 in ZEB2. Upon sumoylation, ZEB2-dependent repression of CDH1 expression is lost, which can be attributed to the concurrent disruption of the ZEB2–CtBP interaction [36].

### 2.5. ZEB1 and ZEB2 Functional Versatility 

Altogether, these data demonstrate that ZEB1 and ZEB2 are highly versatile proteins that can both repress and activate target genes by binding to different cofactors. Moreover, ZEB1 and ZEB2 interact with specific cofactors to perform either overlapping or unique functions in a manner that depends on the cellular context. It is noteworthy that many of the known cofactors of ZEBs are epigenetic remodelling proteins, suggesting an important role for ZEB1 and ZEB2 in shaping the cellular epigenetic landscape in response to intracellular signals and micro-environmental cues.

All interaction partners of ZEB1 and ZEB2 are summarised in Table 1 and Table 2, respectively. The best defined interaction partners in regard to ZEB1 and ZEB2 protein interaction site are illustrated in Figure 1.

## 3. ZEB1 and ZEB2 as Modulators of TGF-β/BMP Signals

### 3.1. TGF-β/BMP Signalling Pathways

The TGF-β and BMP signalling pathways are involved in many cellular processes during embryonic development and physiological homeostasis, as well as in cancer progression. These pathways modulate cell differentiation, stemness, migration, apoptosis, proliferation and senescence, but they are also involved in immune evasion [37,38,39]. In some cellular contexts, these pathways perform synergistic functions, while in others they are considered as mutual antagonists [40]. Canonically, these pathways are activated when extracellular TGF-β and BMP growth factors bind their specific serine/threonine kinase receptors. Consequently, these receptors form transmembrane heterotetramer complexes that can recruit and phosphorylate receptor-mediated SMADs (R-SMADs). SMAD1, SMAD5 and SMAD8 are preferentially used by BMP signalling. SMAD2 and SMAD3, on the other hand, preferentially mediate TGF-β signalling. Upon phosphorylation, these R-SMADs bind the common co-SMAD (SMAD4) and translocate to the nucleus, where they regulate the transcriptional activity of their target genes. Inhibitory SMADs (iSMADs), i.e., SMAD6 and SMAD7, conduct a negative feedback signal [41]. In addition, signalling by TGF-β and BMP can modulate their cellular responses in a SMAD-independent manner. Through this non-canonical pathway, activated TGF-β and BMP receptors activate other signalling pathways, including the phosphatidylinositol 3-kinase (PI3K/AKT), MAPK and Jun N-terminal kinase (JNK)/p38 pathways [40].

### 3.2. ZEB1 and ZEB2 Interact with SMAD Proteins

ZEB1 and ZEB2 can interact with TGF-β and BMP signalling, as both proteins contain a SMAD-binding domain (SBD) (Figure 1). Interestingly, the SBDs of ZEB1 and ZEB2 show little protein sequence similarity. Alignment of ZEB SBDs from different vertebrates allows distinction of ZEB1 SBD from ZEB2 SBD [42]. This suggests that the ZEB transcription factors have evolved to play unique roles in response to the TGF-β and BMP signalling pathways. As already mentioned, ZEB2 interacts with phosphorylated SMADs to form a repressor complex during mesodermal differentiation. More specifically, ZEB2 was shown to interact with SMAD1, SMAD2 and SMAD5 [7]. ZEB1 can also interact to SMAD1 and SMAD2 in HEK293T cells, although less efficient than ZEB2 [43]. Both ZEB2 and ZEB1 interact with activated SMAD3. During osteoblast differentiation, ZEB1 and ZEB2 seem to have antagonistic effects upon TGF-β-mediated signalling. It has been proposed that ZEB1 acts as a transcriptional activator upon binding to SMAD3 while a ZEB2–SMAD3 association acts as a repressor complex [43]. On the other hand, during early neurogenesis in *Xenopus*, both ZEB1–SMAD3 and ZEB2–SMAD3 complexes can act as transcriptional suppressors of BMP-dependent genes. However, suppression by ZEB1 is less efficient than by ZEB2. This is thought to be due to ZEB2 being able to directly bind to SMAD3, while ZEB1 needs to also interact with the co-activators pCAF/p300 (p300/CBP-associated factor; E1A binding protein p300) [44]. Furthermore, binding of ZEB1 to pCAF/p300 via its N-terminal part would displace the corepressor CtBP from ZEB1, turning ZEB1 into a transcriptional activator. It is noteworthy that as ZEB2 can also interact with pCAF/p300 and act as a transcriptional activator, the same regulatory mechanism may also be true for ZEB2 [44,45]. It has been shown in an adult T-cell leukaemia/lymphoma model that ZEB1 counteracts SMAD7-mediated repression of TGF-β signalling. Mechanistically, ZEB1 binds to SMAD7 and additionally recruits a p300/SMAD3 complex to further drive TGF-β signalling [46]. Another study suggested that ZEB2-mediated transcriptional activation of forkhead box protein E3 (FOXE3) during ocular lens development is augmented by SMAD8 association [47].

Despite the different findings on whether ZEB1 and ZEB2 act as antagonistic or synergistic modulators of TGF-β/BMP signalling in osteoblast differentiation and neurogenesis, these findings do not have to be interpreted as contradictory. Differences in the cellular context probably explain the different behaviours of ZEBs towards TGF-β and BMP signalling. This notion is in line with the fact that ZEBs can interact with multiple cofactors depending on the cellular conditions. Hence, it seems that the role of ZEBs in TGF-β and BMP signalling should be studied in specific contexts. In melanoma, ZEB1 and ZEB2 seem to be associated with antagonistic effects on TGF-β signalling, as is described in the following sections.

## 4. ZEBs during Specification from Neural Crest Cells towards the Melanocyte Lineage and in Adult Melanocyte Homeostasis

### 4.1. ZEB1 and ZEB2 in Neural Crest Cells

In vertebrates, the ZEB transcription factors are important throughout the development and differentiation of many cell lineages, such as the haematopoietic lineage [48,49]. The ZEBs have been shown to be crucial mainly during the formation of neural crest cells (NCCs) and their subsequent differentiation into their various derivatives, such as the neurogenic lineage and melanocyte lineage [50,51]. Neural crest cells are motile and give rise to many different cell types, including melanocytes, craniofacial bone and cartilage, peripheral neurons and glial cells, adipose tissue, cardiac smooth muscle cells and secretory adrenal glands. Upon invagination and closure of the neural plate into the neural tube, NCCs delaminate from the neural folds as single cells, which enables them to migrate and subsequently differentiate into their different derivative cell types [51]. This process is a unique and crucial innovation in animal evolution and the formation of vertebrates. The evolutionary duplication and diversification of the ancestral *ZEB* gene, generating *ZEB1* and *ZEB2*, coincides with the evolutionary innovation of neural crest development and specification [42]. Multiple studies using constitutive and conditional *Zeb1-* or *Zeb2*-knockout mice have further proven the importance of these transcription factors in NCC formation. *Zeb1*-null mice die perinatally and are characterised by failure of neural tube closure and skeletal malformations [52]. *Zeb2*-null mice are embryonic lethal with developmental deficiencies from E8.5 onwards, including defects in neural tube closure, neural crest delamination and subsequent migration [53]. Tissue-specific deletion of *Zeb2* in NCCs using *Wnt1-Cre* mice results in defects in craniofacial, heart and peripheral nervous system development [54]. By using *Zeb2*-null mouse embryonic stem cells, it was shown that ZEB2 is essential for exit from the epiblast state and differentiation towards neuroectodermal tissue [55].

EMT is critical for delamination of NCCs, as it drives both detachment and migration. EMT is typically coordinated by the EMT-inducing transcription factors (EMT-TF), namely SNAIL/SLUG (Snail family transcriptional repressor 2), ZEB1/2 and TWIST1 (Twist family BHLH transcription factor 1) [56,57]. Through EMT, epithelial cells lose their epithelial characteristics and gain mesenchymal traits. Typically, CDH1 and other cell–cell interaction proteins are downregulated and cells lose their apicobasal polarity. Thereby, mono- or multi-layered sheets of ordered cells lose their organisation, and the cells can detach as single cells or small groups of several cells. As a result, these cells become highly motile and switch to expressing mesenchymal markers such as N-cadherin (CDH2), fibronectin (FN1) and vimentin (VIM). In a first wave, EMT drives the formation of the neural tube from the neural plates and this is accompanied by a switch from CDH1 to CDH2 expression. This seems to be driven by ZEB2, as CDH1 expression persists in the neuroepithelium of *Zeb2*-null mice [53]. In a second wave of EMT during delamination of the NCCs, another cadherin switch takes place from CDH2 to type II cadherins (cadherin-6/6B/7/11), which have weaker cell–cell adhesion properties [58]. These EMT waves are highly regulated by molecular cues originating from the surrounding embryonic structures. The main signalling pathways involved are the Wnt, fibroblast growth factor (FGF), Notch and BMP pathways [56,57,58].

### 4.2. Neural Crest Cells Migration and Differentiation in the Melanocyte Lineage

The melanocyte and neurogenic lineages are derived almost exclusively from migrating trunk NCCs [50,51]. After detachment, trunk NCCs transiently accumulate at the “migration staging area” and subsequently migrate along two distinct migration routes. Typically, specification into melanocyte lineage precursor cells (melanoblasts or neurogenic progenitor cells) is considered to be closely linked to the migration route taken by the NCCs. Melanoblasts arise from NCCs migrating along a dorsolateral route between the somites and ectoderm, while neurogenic cell populations differentiate from NCCs migrating between the neural tube and somites, known as the ventral migration route. However, melanoblasts have also been found to differentiate from Schwann cell precursors derived from the neural crest and originating from the ventral migration route [59,60]. This points to the existence of a bipotent progenitor cell for glial cells and melanoblasts. Indeed, adult Schwann cells and melanocytes can transit into each other via an intermediate bipotent stem cell in vitro [61]. This also demonstrates the inherent cellular plasticity of melanocyte cells, which is also observed in melanoma cells as will be discussed in subsequent sections.

Melanoblasts can be distinguished from NCCs by their expression of the melanocyte differentiation markers microphthalmia-associated transcription factor (MITF), dopachrome tautomerase (DCT), KIT proto-oncogene (KIT), tyrosinase (TYR) and tyrosinase-related protein 1 (TYRP1) [62]. MITF is the first melanocyte-specific marker expressed in melanoblasts and strong expression is retained in differentiated adult melanocytes. MITF is considered as the master regulator of the melanocyte lineage because it regulates differentiation, cell growth, survival and the synthesis of melanin pigment [63]. MITF expression itself is under the control of different transcription factors, such as cAMP-responsive element-binding protein 1 (CREB), forkhead box protein D3 (FOXD3), paired box gene 3 (PAX3), SRY-box transcription factor 10 (SOX10) and ZEB2 [64,65,66,67,68]. Focusing on ZEB2, it was shown ZEB2 is a driver of MITF expression and downstream melanocyte differentiation markers (e.g., DCT, TYR and TYRP1) in melanoblasts. Moreover, melanocyte-lineage-specific conditional loss of ZEB2 results in a melanoblast migration defect as was shown by a reduction in the number of melanoblasts that can reach the epidermis of the adult skin. This is probably linked to improper differentiation into mature melanocytes, as KIT expression is absent in ZEB2-null melanoblasts. Without KIT expression, melanoblasts are unable to proliferate, which is essential for their migration from dermis to epidermis [68].

Melanoblasts migrate to and colonise three different regions of the adult skin [51]. One group of melanocytes is found in the interfollicular epidermis, where they differentiate into adult melanocytes and are responsible for skin pigmentation. A second group colonises two regions of the dermal hair follicle that have distinct micro-environments. A first subpopulation inhabits the lower part of the hair follicle close to the dermal papilla (bulb area), where they immediately differentiate into mature melanocytes signalling to contribute to hair pigmentation during the first hair cycle. A second subpopulation occupies the “bulge area” of the hair follicle, which is a specialised stem cell niche. Thus, instead of differentiating into mature melanocytes, this subpopulation is present as undifferentiated melanocyte stem cells characterised by weak expression of the melanocyte differentiation markers MITF, TYR and TYRP1 [69]. In adult skin, the hair follicle undergoes continuous hair cycles consisting of three phases [70]. The cycles start with a growth phase coupled to proliferation and differentiation of melanocyte stem cells into mature melanocytes (anagen). This is followed by regression of the hair follicle and apoptosis of the differentiated melanocytes but not melanocyte stem cells (catagen). Afterwards, the hair follicle goes into a resting phase (telogen) in which the melanocyte stem cells are kept quiescent until the next anagen. Upon every hair cycle, the melanocyte stem cells are “activated” to replenish the growing hair follicle with new mature melanocytes.

### 4.3. Balance between ZEB1 and ZEB2 in Melanocyte Homeostasis at the Hair Follicle

**Figure 2:**
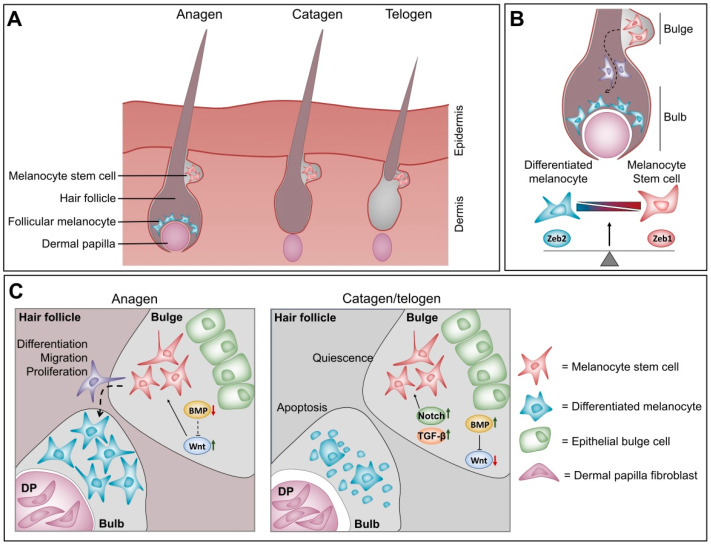
The intrinsic balance between ZEB1 and ZEB2 plays a role in melanocyte homeostasis during the hair cycle (**A**) Schematic representation of the hair cycle. Differentiated follicular melanocytes are only present during anagen phase while melanocyte stem cells are retained throughout the different phases of the hair cycle. (**B**) Strong ZEB1 expression is associated with a quiescent melanocyte stem cell state in the bulge area of the hair follicle (red coloured cells). Strong ZEB2 expression drives melanocyte differentiation and well-differentiated melanocytes are typically present in the bulb area of the hair follicle (blue coloured cells). (**C**) A balance between different micro-environmental signalling cues modulates melanocyte homeostasis during the hair cycle. These signalling cues hypothetically function by influencing the ZEB expression balance in melanocytes.

A balance between different micro-environmental cues at the bulb and bulge areas seems to regulate this periodic activation of the hair cycle. During the catagen and telogen phases, active Notch and TGF-β signalling combined with suppression of Wnt signalling by active BMP signalling at the bulge region seem to keep the melanocyte stem cells quiescent (Figure 2) [71,72,73,74]. Upon activation of the anagen phase, CD133-positive cells at the dermal papilla secrete WNT-ligand, which activates Wnt signalling at the bulge area. This increased Wnt signalling shifts the balance from a quiescent melanocyte stem cell state to an asymmetric proliferating melanocyte stem cell state. Part of these cells then migrate to the bulb area of the hair follicle and differentiate into mature melanocytes to contribute to hair pigmentation (Figure 2) [75,76]. A balance between the ZEB transcription factors also seems to modulate melanocyte differentiation. As mentioned above, ZEB2 is important not only for melanoblast migration but also for their proper differentiation into mature melanocytes [68]. This was observed by the loss of hair pigmentation in the melanocyte lineage-specific, conditional *ZEB2^KO^* adult mice. These mice showed that the *ZEB2^KO^* melanocyte stem cells present in the bulge could still migrate to the bulb area upon activation of the hair cycle. However, these cells were unable to fully differentiate into mature, pigment-producing melanocytes. These *ZEB2^KO^* immature melanocyte cells expressed less MITF compared to cells from *ZEB2* wild type mice. Moreover, transcriptional analysis of in vitro ZEB2 knock-down experiments in primary melanocytes revealed that this ZEB2-dependent decrease in MITF coincides with upregulation of ZEB1. Taken together, this suggests an antagonistic function of ZEBs in melanocyte homeostasis, where ZEB2 and MITF drive a differentiated melanocyte cell state, while ZEB1 is associated with a more melanocyte stem cell state. ZEB1 and ZEB2 can function in response to Wnt, Notch and TGF-β signalling in other cellular contexts. It is reasonable to think that this is also the case in melanocyte homeostasis. Given these observations, it is plausible that Wnt signalling cooperates with ZEB2 function to drive MITF expression and differentiation, whereas stemness driven by Notch and TGF-β is associated with ZEB1 activity.

## 5. ZEB1 and ZEB2 Induce EMT-Driven Cellular Plasticity during Carcinoma Progression

Research on the ZEB transcription factors in melanoma is limited. However, these factors have been studied extensively as drivers of carcinoma progression. A thorough overview of the roles of ZEBs in carcinoma is outside the scope of this review and has been reviewed recently by several research groups [77,78,79,80,81,82]. However, as the roles of ZEBs show some similarities as well contradictory functions in relation to carcinoma progression, it is appropriate to briefly summarise recent findings on this topic. Carcinoma arises from epithelial tissues, where aberrant expression of the ZEB transcription factors (and other EMT TFs) reactivates the embryonic EMT process. As in NCC development, this leads to downregulation of CDH1 and other epithelial cell–cell interaction proteins, while favouring the expression of mesenchymal markers such as CDH2. Classically, this expression switch in carcinoma is associated with loss of intercellular adhesion and reorganisation of the cytoskeleton. The morphology of carcinoma cells changes to more mesenchymal-like spindle-shaped cells with decreased proliferative capacity but increased migratory capacity. As such, ZEB1 and ZEB2 are mainly considered as drivers of tumour cell invasion into distant tissues. Once they arrive at these tissues, tumour cells need to revert to an epithelial phenotype through mesenchymal-to-epithelial transition (MET) in order to colonise the site and proliferate [83,84].

This classical binary view of EMT that cells are present either in the epithelial or the mesenchymal state has become obsolete. It has become clear that full-blown EMT does not readily occur during carcinoma progression. Rather, carcinoma cells can be present in a spectrum of cellular states. They can be present within a tumour expressing both epithelial and mesenchymal markers, and they can reversibly switch between different cellular states ranging from the extreme epithelial to the extreme mesenchymal state. This reversible plastic process, known as epithelial–mesenchymal plasticity (EMP) [85], explains how certain carcinoma cells within a tumour can express both epithelial and mesenchymal markers [79,82]. How the presence of different cell states influences the metastatic potential of tumour cells has not been determined. However, it is clear that this cellular plasticity is regulated by the EMT TFs and is the main driver of intra-tumour heterogeneity, which is linked to therapy resistance. Indeed, EMT TFs influence not only the proliferative and invasive capacities of carcinoma cells but are also important in other EMP-related processes, including cell differentiation, cell state transition, cancer stem cell formation, survival and senescence, therapy resistance, immune evasion and shaping of the micro-environment [77]. Interestingly, ZEB-driven cellular plasticity is under the control of similar micro-environmental signals, which also modulate their expression during development. These are the TGF-β/BMP, Wnt, Notch and Sonic hedgehog (Shh) signalling pathways. In addition, hormones, hypoxia, nuclear factor kappa-light-chain-enhancer of activated B cells (NF-κB) signalling and crosstalk with other EMT-inducing transcription factors further modulate the expression of ZEB1 and ZEB2 [5,80]. As already suggested by the many epigenetic remodelling proteins that bind to the ZEBs in different carcinoma contexts (previous section), their functions are not only limited to direct transcriptional repression or activation of target genes. Indeed, mounting evidence suggests that ZEB1 and ZEB2 are also important epigenetic factors recruiting cofactor complexes to drive epigenetic remodelling during cellular plasticity (recently reviewed in [78]).

As described above, melanocytes are not epithelial cells but can be regarded as a product of EMT during NCC specification. Intrinsically, EMT does not occur in melanoma cells. Nonetheless, ZEB-driven cellular plasticity also plays an important role in melanoma. It is likely that the signalling cues and roles of ZEBs described here are also important in melanoma “phenotype switching”.

## 6. The “Phenotype-Switching” Model for Melanoma Heterogeneity

### 6.1. The Reversible “Phenotype-Switching” Model

During the last decades, three different cancer progression models have been proposed to try to cope with the therapy resistance of melanoma induced by intra- and inter-tumour heterogeneity. The “clonal evolution” model states that (epi)genetic mutations occur serially in all tumour cells. Tumour cells that gain an advantage due to such changes can out-compete others through natural selection [86]. Certain tumour clones gain a mutation that makes them resistant to a specific therapy while other clones remain sensitive. In the “cancer stem cell” model, tumours consist of different cell populations. The majority of cancer cells are more differentiated and highly proliferative, while a minority of cells behave as quiescent stem-like cells that drive tumour metastasis [87]. The current therapies that target proliferation pathways target the highly proliferative tumour cells. However, the quiescent stem cell-like population escapes and drives remission. Even though both models describe the presence of subpopulations with different sensitivities to therapy within a tumour, they cannot fully explain the mechanisms by which melanoma cells respond to the current therapies. Both models describe changes within the cancer cell that drive it to a certain phenotypic state as a hierarchical one-way event. However, as melanoma cells show high cellular plasticity that enables them to reversibly change between different phenotypic states, these above-described static models fall short of explaining the influences modulating this reversible plasticity.

The “phenotype-switching” model, proposed by Hoek and Goding, was the first model to explain this reversibility [88]. This model states that the influence of cell-extrinsic signals, in combination with its cell-intrinsic changes, impose the melanoma cell state. Epigenetic changes and reversible transcriptional and post-translational modifications determine the cellular phenotype state upon responding to certain extracellular cues. However, the intrinsic genetic mutations influence how the cancer cells respond to specific signalling cues. Micro-environmental signals drive the reversible phenotypic switch between a more proliferative and a highly invasive phenotype. Moreover, this phenotypic reprogramming induced by the micro-environment converges onto the expression state of MITF [89]. In vitro, two distinct expression signatures have been observed: MITF^high^-expressing, differentiated and proliferative melanoma cells versus slow growing, MITF^low^-expressing, mesenchymal-like melanoma cells that are more invasive. The MITF^high^ expression signature was associated with the expression of the melanogenesis pathway (DCT, TYR and premelanosome protein (PMEL)), while the MITF^low^-expressing cells expressed more TGF-β-regulated markers (e.g., CDH1) and stem cell markers (e.g., CD271 and nerve growth factor receptor; NGFR). Transplant experiments in vivo showed that both populations could generate heterogeneous tumours comprising both subpopulations [90], demonstrating the reversible cellular plasticity of the “phenotype-switching” model. This model was further validated by additional studies [68,91,92,93,94,95]. Similar to carcinoma cell plasticity, melanoma cell plasticity has also been associated with cellular properties other than proliferation, cell differentiation state and invasion, such as stemness, survival, therapy resistance and immune evasion [96,97,98,99,100,101,102,103].

### 6.2. Implications of “Intermediate” Melanoma Cell States for Therapy Resistance

Single-cell platforms have further made it possible to study the complexity of melanoma heterogeneity and cell plasticity. Single-cell RNA-seq has revealed that primary melanomas and melanoma metastases also contain cells expressing markers of both the MITF^high^ and the MITF^low^ expression signatures [104,105]. This observation demonstrates that there is no restriction of only two well-defined subpopulations in a melanoma tumour. Most likely, melanoma cells are present in a spectrum of cellular states ranging between the MITF^low^ and MITF^high^ expression states within a single melanoma tumour, similar to EMP. It still has to be determined whether these “intermediate” cellular states are transient or stable and which of them has a functional impact on melanoma tumour behaviour. Two recent studies have defined such a stable “intermediate” state [106,107]. They describe a neural crest stem cell (NCSC)-like melanoma state that expresses some melanocyte differentiation markers, e.g., MITF and SOX10, as well as mesenchymal-like markers (e.g., FN1 and S100 calcium binding protein A16 (S100A16)), immune-related markers (interferon-induced transmembrane protein 3 (IFITM3) and major histocompatibility complex, class I, B (HLA-B)) and neural crest stem-cell makers (Nestin (NES) and MIA SH3 domain containing (MIA)). Moreover, this NCSC-like intermediate state seems to be regulated by transcription factors that are known drivers of the differentiated state (e.g., MITF and SOX10) and of the invasive state (e.g., Jun proto-oncogene (JUN) and FOS like 2 (FOSL2)). Next to these, some transcription factors were found that are specifically active in this intermediate state, including early growth response 3 (EGR3), retinoid X receptor gamma (RXRG) and nuclear factor of activated T-cells 2 (NFATC2) [106]. Interestingly, this RXRG-driven NCSC-like state seems to drive therapy resistance to BRAF/MEK inhibition in BRAF^V600E/K^ mutant xenografts from patients. Upon treatment with dabrafenib–trametinib combination therapy, these xenografts first respond well and shrink but acquire resistance over time, resulting in minimal residual disease (MRD). Single-cell RNA-seq analysis of these MRDs identified a cluster of RXRG-driven NCSC-like melanoma cells. However, treating these xenografts with dabrafenib–trametinib combined with RXRG inhibition mitigates accumulation of NCSC-like cells in the MRD and delays development of therapy resistance [107]. Taken together, these NCSC-like melanoma cells are characterised by increased migratory capacity compared to extremely differentiated melanoma cells, but to a lesser degree than extremely invasive melanoma cells, and by increased resistance to MAPK inhibitors (MAPKi) therapy [106,107]. A similar observation was made in another recent study, but in that case four distinct subpopulations were defined instead of three [108]: an extremely differentiated state, a gradual less differentiated state, a gradual still lesser differentiated/NCSC-like state and an extremely dedifferentiated mesenchymal-like state. Moreover, this study suggested that melanoma cell dedifferentiation was a transient response to both MAPKi and immunotherapy that enabled the cells to acquire resistance. Interestingly, an inverse correlation was shown between differentiation status and sensitivity to ferroptosis. Taken together, these studies show that melanoma cells can switch to an “intermediate” NCSC-like state that contributes to resistance against the current therapies. Moreover, they show the importance of targeting intermediate subpopulations to treat melanoma patients more effectively.

## 7. ZEB1 and ZEB2 in Melanoma Phenotype Switching

### 7.1. ZEBs and Melanoma Differentiation/Proliferation vs Invasion

As mentioned above, the ZEB transcription factors are generally thought to perform similar functions in epithelial cancer context and have mainly been studied as drivers of EMP. In melanoma context, the roles of ZEB1 and ZEB2 seem to be antagonistic and to function in line with the “phenotype-switching” model. Two research groups have independently shown that strong ZEB2/SLUG expression in primary melanomas is a positive prognostic factor for melanoma patients. Similar to their observed roles in melanocyte homeostasis, the ZEB transcription factors seem to have opposing roles in melanoma that converge at the expression level of MITF. Strong ZEB2/SLUG expression in melanoma is associated with high levels of MITF and downstream melanocyte differentiation markers. On the other hand, a strong ZEB1/TWIST1 expression signature correlates with low levels of MITF and downstream differentiation markers and gain of mesenchymal markers and invasiveness. Ectopic expression of ZEB2 can reverse the switch from a ZEB1^high^/MITF^low^, invasive cell state to a ZEB2^high^/MITF^high^ differentiated melanoma cell state [68,92]. Interestingly, it seems that increased MAPK signalling can trigger this switch from a ZEB2 to a ZEB1 expression signature. This mechanistically links the typical melanoma oncogenic driver mutations to the “phenotype-switching” model. Constitutively active BRAF^V600E^ or NRAS^Q61K^ expression drives the ZEB1^high^/MITF^low^ cellular state via the AP-1 transcription factor, FRA1 [92]. Furthermore, a recent study shows that ZEB2 is not only associated with but actually acts as an essential driver of melanoma development and growth in vivo. Ablation of ZEB2 in a NRAS^Q61K^-driven mouse model delays melanoma initiation and attenuates nevus and melanoma outgrowth. Moreover, the observed tumours in this model originated from melanoma cells that escaped ZEB2 ablation. ZEB2 overexpression in the same genetic background drives the formation of macro-metastasis, whereas only micro-metastases are observed in ZEB2 wild type mice. Next to this, ZEB2 actively modulates phenotype switching in both mouse and human. In vivo ZEB2 overexpression and in vitro ZEB2 knock-down experiments show that ZEB2 drives the proliferative/differentiated melanoma gene signature while suppressing the invasive gene signature [109].

### 7.2. ZEBs and Melanoma Stemness

In melanocyte homeostasis, a ZEB2^low^/MITF^low^ plus ZEB1^high^ expression signature is linked to a melanocyte stem cell phenotype [68]. This expression signature also seems to drive stemness in melanoma cells. ZEB1 has been proposed to be important for maintenance of the cancer stem cell (CSC) properties in the presumed CD133^+^/CD44^+^ cancer stem cell subpopulation in B16-F10 melanoma cells [110,111]. Here, knock-down of ZEB1 in B16-F10 CD133^+^/CD44^+^ CSCs results in significantly reduced clonogenicity, proliferation and migratory properties in vitro and reduced tumour growth and lung tumour metastasis in vivo. Additional evidence for a ZEB1 role in melanoma CSC maintenance comes from another study in which the neural crest cell marker Msh homeobox 1 (MSX1) was shown to reprogram differentiated melanocytes to more invasive, NCSC-like melanocytes that can differentiate into other NCSC derivatives, such as neuronal cells. This cell state reprogramming coincides with an increase in ZEB1 and a decrease in ZEB2 and MITF expression [112]. Moreover, that study has shown that reactivation of notch receptor 1 (NOTCH1) signalling fully reprogrammed differentiated melanocytes into multipotent NCSC-like cells. Further, MSX1 was also significantly upregulated in these NOTCH1-induced NCSC-like cells [113]. Taken together, this suggests a potential NOTCH1–MSX1–ZEB1 axis that drives melanoma stemness. These studies also further support the potential link between Notch signalling and ZEB1 in maintaining the melanocyte stem cells in a quiescent state at the bulge region of the hair follicle during the telogen phase of the hair cycle. Remarkably, these data potentially link ZEB1 to the intermediate NCSC-like cell state observed in the single-cell RNA-seq studies on phenotype switching mentioned in the previous section. We used the online tool SCOPE (http://scope.aertslab.org/) to examine the single-cell RNA-seq data from [106] for ZEB expression. From these data, it is clear that ZEB1 is strongly expressed in the extremely invasive cell state (SRY-box transcription factor 9 (SOX9) positive cells) but is also expressed in the intermediate NCSC-like cells (EGR3, RXRG positive), suggesting a role of ZEB1 in the observed NCSC-like state. Interestingly, some of these NCSC-like cells still express ZEB2 and MITF, indicating that this is an intermediate cell state originating from the ZEB2/MITF-positive differentiated cell state.

### 7.3. ZEBs and Melanoma Therapy Resistance

Strong ZEB1 expression is also associated with increased therapy resistance in melanoma cells. Both MAPKi-resistant BRAF^V600E^-mutated melanoma cell lines and tumour biopsies obtained from human MAPKi-resistant BRAF^V600E^ patients express high levels of ZEB1 but low levels of ZEB2 and MITF. In vitro overexpression of ZEB1 in therapy-naive cells results in downregulation of MITF, upregulation of cancer stem cell markers (e.g., NGFR and lysine demethylase 5B, JARID1B) and mesenchymal markers (e.g., VIM), and increased resistance to MAPKi. ZEB1 knock-down in MAPKi-resistant cell lines sensitised these cells to MAPKi treatment, shown by a decrease in cell viability upon MAPKi treatment. Concomitantly, these cells harboured a ZEB2^high^/MITF^high^/NGFR^low^ expression signature. Finally, in silico data linked intrinsic MAPKi-resistance to a ZEB1^high^/ZEB2^low^/MITF^low^ signature, whereas MAPKi sensitivity was associated with a ZEB1^low^/ZEB2^high^/MITF^high^ profile [114]. Whether ZEB1 actively drives therapy resistance or otherwise resistance is due to the suppression of ZEB2/MITF is not yet clear. Different articles have reported a dual role for MITF in MAPKi resistance. One report states that high MITF levels contributed to MAPKi resistance [115], whereas two other reports identified weak MITF expression as predictive of intrinsic MAPKi resistance [98,99]. These findings favour the idea that increased ZEB1 expression rather than loss of MITF expression is the driver of MAPKi resistance. Remarkably, it has been shown that therapy resistant ZEB1^high^ melanoma cells are metabolically dependent on the glutathione peroxidase 4 (GPX4)-driven lipid-peroxidase pathway. Consequentially, these melanoma cells are vulnerable to ferroptotic cell death induced by inhibition of this lipid peroxidase pathway [116]. This observation coincides with the observed ferroptosis sensitivity of the intermediate NCSC-like state, suggesting a role for ZEB1 in driving this phenotype switch from a differentiated state [108]. Therefore, targeting ZEB1 could prove to be a promising approach to reverse the NCSC-like state towards a more differentiated state and/or decrease the associated therapy resistance. However, it first has to be determined whether ZEB1 actually drives this phenotypic switch, as most studies up until now have rather shown a strong association with this phenotype.

## 8. Micro-Environmental Cues Regulate the Reversible Balance between ZEB1 and ZEB2

Several signalling pathways have been shown to modulate melanoma phenotype switching, including TGF-β and WNT signalling, hypoxia and inflammation, among others [89,117,118]. These signalling cues have also been shown to modulate ZEB proteins expression in the context of melanocyte homeostasis and/or carcinoma plasticity. As such, it is to be expected that some of these micro-environmental associated signalling pathways regulate melanoma plasticity through modulation of the balance between ZEB2 and ZEB1. 

### 8.1. TGF-β/Shh Signalling

Next to the already mentioned potential NOTCH1–ZEB1 signalling axis, the TGF-β signalling pathway has also been shown to be a major driver of a melanoma phenotype switch to a more ZEB1^high^ invasive state [89,119]. Moreover, two papers suggest a mechanistic link between TGF-β signalling and the Shh pathway in driving melanoma invasion in a ZEB1-dependent manner. Induction of TGF-β signalling induces the expression of GLI family zinc finger 2 (GLI2), which in turn downregulates MITF while upregulating ZEB1 expression. This leads to the formation of a GLI2-ZEB1 complex that is associated with weaker CDH1 expression [120]. Next to this, GLI family zinc finger 1 (GLI1) is proposed to be important for maintaining the invasive properties of melanoma cells in a ZEB2/MITF-independent manner but most likely ZEB1-dependent manner. Knock-down of GLI1 in the B16F10 mouse melanoma cell line resulted in a reduced invasive capacity in vitro and decreased lung metastasis ability in vivo. Moreover, knock-down of GLI1 lead to a decreased *Zeb1* mRNA expression but did not change *Zeb2/Mitf* mRNA expression [121]. Taken together, these papers suggest that activation of TGF-β signalling is important during the initial downregulation of MITF and the melanoma differentiation signature. Consequently, this drives a more invasive expression signature by increased expression of ZEB1 and of modulators of the Shh pathway, such as GLI2. Once established, this invasive melanoma signature is then stabilised via GLI1 and ZEB1 signalling. It is noteworthy that the above-mentioned ZEB2/MITF independency was determined by checking RNA expression levels. However, a recent study has shown that upon activation of TGF-β signalling, *Zeb2* mRNA levels stay the same but ZEB2 protein levels clearly drop [109]. Therefore, ZEB2 protein levels should be evaluated to furthermore confirm the proposed ZEB2-independent functioning of GLI1 in melanoma invasion.

### 8.2. Hypoxia

Hypoxia is another extracellular signal that promotes the conversion from a differentiated to an invasive state in melanoma. Upon activation of hypoxia inducible factor 1 alpha (HIF1A) by hypoxic conditions, MITF expression is downregulated, with a consequent increase in metastatic potential [122]. Moreover, expression of ZEB1 and FN1 is increased while expression of melanocyte differentiation marker Melan-A (MLANA) is downregulated, suggesting an actual phenotype switch induced by HIF1A [123]. Another study further demonstrates that mainly a subset of FN1^high^/MITF^low^ melanoma cells express ZEB1, which is in line with the expression of the hypoxic marker hypoxia inducible factor 2 subunit alpha (HIF2A) and stem cell markers NGFR and JARID1B [93].

### 8.3. Hippo Signalling Pathway

The more recently discovered Hippo signalling pathway has been linked to cancer progression. This pathway is typically activated by changes in cell polarity, energy stress, transmembrane GPCRs and stiffness of the extracellular matrix [124]. The Hippo signalling pathway effector YAP has been shown to drive ZEB1 expression in breast cancer. Moreover, YAP and ZEB1 directly interact with each other to drive a common ZEB1/YAP expression signature. This signature indicates poor relapse-free survival, therapy resistance and increased metastatic risk [35]. Similarly, in melanoma, high YAP and ZEB1 expression is associated with a more invasive gene expression signature and ectopic expression of YAP induces melanoma invasion both in vitro and in vivo [125]. These data suggest a plausible active YAP/ZEB1 complex in melanoma as well although this complex should still be verified experimentally.

### 8.4. EMT-Associated Transcription Factors

Different EMT-associated transcription factors, of which expression is also modulated by the micro-environment, typically influence each other’s activity in a number of cancer contexts. A high ZEB2/SLUG expression signature is associated with a more differentiated melanoma state and better patient prognosis [68,92]. However, contradictory results were observed in one study that suggests that SLUG drives the switch towards a ZEB2^low^/ZEB1^high^ phenotypic state [126,127]. In vitro experiments have shown that overexpression and knock-down of SLUG, respectively, increases and decreases ZEB1 expression. Moreover, SLUG and ZEB1 were both found to increase the migratory capacities of human melanoma cell lines in vitro [126]. Another study showed that in vivo knock-down of SLUG resulted in decreased melanoma metastasis potential. However, this result does not necessarily imply that SLUG is important for in vivo invasion. It is more likely that SLUG drives melanoma metastasis by promoting secondary tumour outgrowth in a similar manner as ZEB2 does [127]. It is noteworthy that the mentioned contradictory results were obtained from in vitro data only and this may explain the discrepancy compared to the other studies using in vivo data as well.

**Figure 3:**
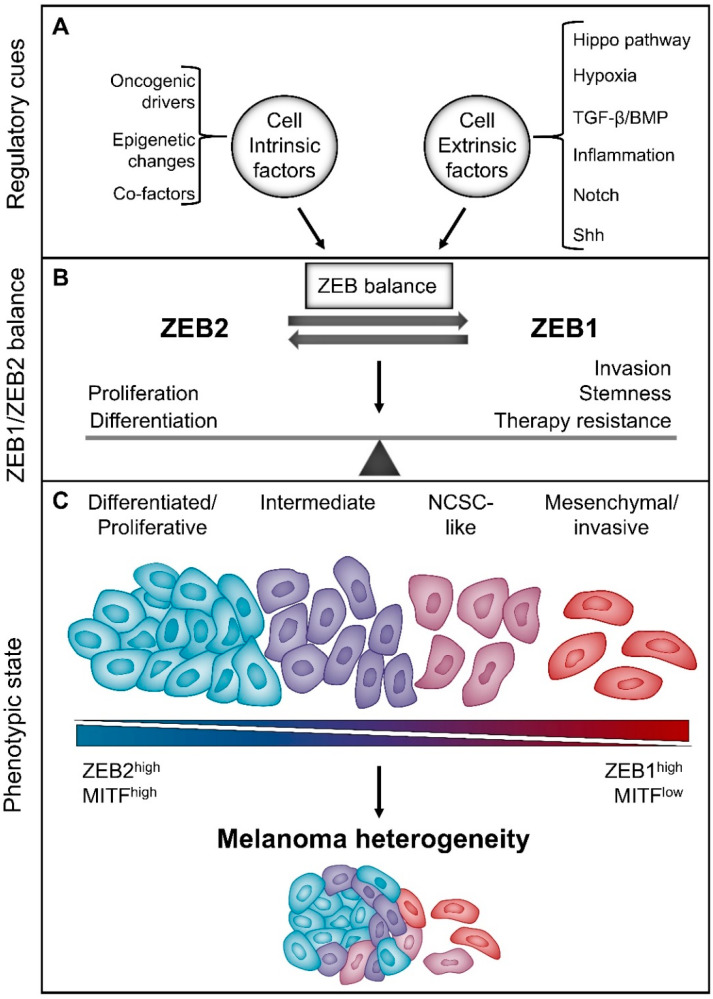
The intrinsic balance between ZEB1 and ZEB2 modulates melanoma phenotype switching, which drives melanoma heterogeneity. (**A**) Melanoma heterogeneity is steered by cell intrinsic factors and cell extrinsic cues that regulate the expression balance between ZEB1 and ZEB2. (**B**) ZEB2 is a regulator of differentiation/proliferation while ZEB1 is associated with cancer stemness, migration/invasion and therapy resistance. (**C**) A spectrum of melanoma cell states exists dependent on the reversible balance between a ZEB2/MITF^high^ expression signature and a ZEB1^high^ expression signature. This spectrum of melanoma cell states contributes to melanoma heterogeneity.

The regulatory cues modulating the balance between ZEB1 and ZEB2 expression and the roles of ZEB1 and ZEB2 in melanoma context are summarised in Figure 3.

## 9. Conclusions and Perspectives: Targeting ZEB1 and ZEB2 to Modulate Melanoma Phenotype Switching

Studies on melanocyte development and hair follicle homeostasis teach us that melanocytes are intrinsically plastic cells that can switch their differentiation status depending on micro-environmental signals. The “phenotype-switching” model extrapolates this cellular plasticity to the melanoma context and implies that most melanoma cells can reversibly change between different phenotypes. Melanoma cells are present in a spectrum of different states ranging from the extremely differentiated melanocyte-like state to the dedifferentiated/invasive mesenchymal-like state. Moreover, at least one stable intermediate state harbouring neural crest stem cell-like markers has been defined. More significantly, this cellular plasticity has important implications for tumour behaviour. It seems that both partly and fully dedifferentiated subpopulations can drive tumour invasiveness, therapy resistance and immune invasion.

Due to melanoma being a highly aggressive tumour, many patients are diagnosed at the metastasis stage. Most of these patients are initially responsive to the current therapies. However, acquired resistance and relapse are common. To minimise relapse after therapy, it is essential to target the (partly) dedifferentiated subpopulations. A potential strategy is to therapeutically switch these subpopulations back to the differentiated melanocyte-like state, in combination with the current melanoma therapies, to eliminate the therapy-resistant tumour cells.

ZEB1 and ZEB2 play important roles in driving melanocyte and melanoma plasticity. ZEB2 drives the proliferative and differentiated melanocyte state through MITF, while ZEB1 seems to drive loss of MITF and dedifferentiation and is associated with NCSC-like properties and therapy resistance. Modulating the balance between the ZEBs in favour of ZEB2 could have an interesting therapeutic potential for controlling the melanoma phenotypic state. Understanding which micro-environmental cues and cell-intrinsic regulators determine this ZEB balance in melanoma can lead to potential novel therapeutic strategies. More research should be done to better understand the complex regulatory network modulating this ZEB balance. A potential therapeutic strategy is suppression of the Notch and TGF-β signalling pathways as well as hypoxia and YAP signalling, all of which seem to shift the balance in favour of ZEB1 expression.

Rather than targeting the upstream regulation of ZEBs, another therapeutic strategy could be to block their downstream activities. The ZEB transcription factors have been shown to function both as direct transcriptional regulators and as epigenetic modulators by recruiting diverse co-factors, depending on the cellular context. In this way, they can perform pleiotropic functions. However, how these transcription factors mechanistically perform these functions specifically in melanoma has to be elucidated. By disrupting their interaction with specific cofactors, we could potentially suppress specific activities of either ZEB1 or ZEB2. For example, ZEB2 is associated with both the favourable differentiated melanocyte-like state and with cell proliferation and switching from a ZEB1 to a ZEB2 state involves the risk of promoting secondary tumour outgrowth. Suppressing the ZEB2-associated proliferation but not the pro-differentiation function could prove beneficial in combination with current therapies or the above-mentioned strategy.

## Figures and Tables

**Table 1:** Summary of known interaction partners of ZEB1.

Protein/Complex	Complex	Region of Interaction	Target Gene	Activity	Regulation	Cellular Context	Refs
POU2F1	Na	HD	Na	Na	Na	*Rat* ZEB1	8
CtBP1/2	CtBP repressor complex	CID	CDH1 & GH	Repression	Transcriptional & Epigenetic	Breast carcinoma & pituitary differentiation	12,14,15
HDAC1/2	Na	Not defined	CDH1	Repression	Epigenetic	Pancreatic cancer	21
SMARCA4	SWI/SNF	N-terminal	CDH1	Repression	Epigenetic	HEK293T cells	23
DNMT1	Na	SBD	CDH1	Repression	Epigenetic	Mammary gland/Breast cancer	24
hTERT	Na	Not defined	CDH1	Repression	Transcriptional	Colorectal cancer	25
SIRT1	Na	Not defined	CDH1	Repression	Epigenetic	Prostate cancer	27
SET8	Na	Not defined	CDH1	Repression	Epigenetic	Prostate cancer	28
TIP60	Na	N-terminal half	CD4	Repression	Epigenetic	Jurkat/Hela cells	29
NC2	Na	Between HD & CZF	In vitro reporter constructs	Repression	Transcriptional	Hela cells	30
NuRD complex	NuRD	Not defined	TBC1D2b	Repression	Epigenetic	Lung cancer	32
SRF	SMAD3	NZF & CZF	α-actin	Activation	Transcriptional	Smooth muscle cell differentiation	33
LMO2	Na	N-terminal	ZEB1	Represses ZEB1 transcription/ activity	Transcriptional/ post-translational	T-cell leukemia	34
YAP	Na	N-terminal & C-terminal	Common ZEB1/YAP target gene set	Activation	Transcriptional	Breast cancer	35
PC2	Na	Lys-347 & Lys-774	ZEB1	Sumoylates and represses ZEB1 activity	Post-translational	COS-1 cells	36
pCAF/p300	SMAD3	N-terminal	Xbra/Gata2/Msx1	Activation	Transcriptional	Neurogenesis	44,45
SMAD1	Na	SBD	Na	Na	Na	HEK293T cells	43
SMAD2	Na	SBD	Na	Na	Na	HEK293T cells	43
SMAD3	SRF & Na & pCAF/p300	SBD	α-actin & BMP- /TGF-β-signalling & Xbra/Gata2/Msx1	Activation & Repression	Transcriptional	Smooth muscle cell - & Osteoblast differentiation & Neurogenesis	33,43,44,45
SMAD7	Na	SBD	SMAD7	Represses SMAD7 activity	Post-translational	T-cell leukemia/lymphoma model	46

**Table 2:** Summary of known interaction partners of ZEB2.

Protein/Complex	Complex	Region of Interaction	Target Gene	Activity	Regulation	Cellular Context	Refs
CtBP1/2	CtBP repressor complex	CID	BMP4	Repression	Transcriptional & Epigenetic	Neurulation	13
LSD1	Na	Not defined	CD11b	Repression	Epigenetic	T-ALL	20
NuRD complex	NuRD	NIM	CDH1	Repression	Epigenetic	HEK293T cells	31
PC2	Na	Lys-391 & Lys-866	ZEB2	Represses ZEB2 activity	Post-translational	COS-1 cells	36
pCAF/p300	SMAD3	N-terminal	Xbra/Gata2/Msx1	Activation	Transcriptional	Neurogenesis	44,45
SMAD1	Na	SBD	Xbra2	Repression	Transcriptional	Mesoderm differentiation	7
SMAD2	Na	SBD	Xbra2	Repression	Transcriptional	Mesoderm differentiation	7
SMAD3	Na & pCAF/p300	SBD	BMP/TGF-β-signalling & Xbra/Gata2/Msx1	Repression & Activation	Transcriptional	Osteoblast differentiation & Neurogenesis	43,44,45
SMAD5	Na	SBD	Xbra2	Repression	Transcriptional	Mesoderm differentiation	7
SMAD8	Na	SBD	Foxe3	Activation	Transcriptional	Ocular lens development	47
